# Ease and equity of access to free DR-TB services in Nigeria- a qualitative analysis of policies, structures and processes

**DOI:** 10.1186/s12939-020-01342-w

**Published:** 2020-12-10

**Authors:** Charity Oga-Omenka, Florence Bada, Aderonke Agbaje, Patrick Dakum, Dick Menzies, Christina Zarowsky

**Affiliations:** 1 The School of Public Health of the University of Montreal (ÉSPUM), 7101, Parc avenue, 3rd floor, Montreal, Quebec H3N 1X9 Canada; 2 Centre de recherche en santé publique, Université de Montréal (CReSP), Montreal, Canada; 3 McGill University International TB Centre, Montreal, Quebec Canada; 4 International Research Center of Excellence, Institute of Human Virology Nigeria, Abuja, Nigeria; 5 Department of Epidemiology and Biostatistics, McGill University, Montreal, Canada; 6 School of Public Health, University of the Western Cape, Cape Town, South Africa

## Abstract

**Introduction:**

Persistent low rates of case notification and treatment coverage reflect that accessing diagnosis and treatment for drug-resistant tuberculosis (DR-TB) in Nigeria remains a challenge, even though it is provided free of charge to patients. Equity in health access requires availability of comparable, appropriate services to all, based on needs, and irrespective of socio-demographic characteristics. Our study aimed to identify the reasons for Nigeria’s low rates of case-finding and treatment for DR-TB. To achieve this, we analyzed elements that facilitate or hinder equitable access for different groups of patients within the current health system to support DR-TB management in Nigeria.

**Methods:**

We conducted documentary review of guidelines and workers manuals, as well as 57 qualitative interviews, including 10 focus group discussions, with a total of 127 participants, in Nigeria. Between August and November 2017, we interviewed patients who were on treatment, their treatment supporter, and providers in Ogun and Plateau States, as well as program managers in Benue and Abuja. We adapted and used Levesque’s patient-centered access to care framework to analyze DR-TB policy documents and interview data.

**Results:**

Thematic analysis revealed inequitable access to DR-TB care for some patient socio-demographic groups. While patients were mostly treated equally at the facility level, some patients experienced more difficulty accessing care based on their gender, age, occupation, educational level and religion. Health system factors including positive provider attitudes and financial support provided to the patients facilitated equity and ease of access. However, limited coverage and the absence of patients’ access rights protection and considerations in the treatment guidelines and workers manuals likely hampered access.

**Conclusion:**

In the context of Nigeria’s low case-finding and treatment coverage, applying an equity of access framework was necessary to highlight gaps in care. Differing social contexts of patients adversely affected their access to DR-TB care. We identified several strengths in DR-TB care delivery, including the current financial support that should be sustained. Our findings highlight the need for government’s commitment and continued interventions.

## Introduction

Nigeria has overlapping high burdens of tuberculosis (TB), drug resistant tuberculosis (DR-TB) and HIV, according to the World Health Organisation (WHO) [1]. However, in 2018, the country of 198 million people had one of the lowest global TB case detection rates at 15%. Only about 11 and 9% of estimated DR-TB cases were notified and initiated on treatment, respectively [1]. This highlights major difficulties in accessing DR-TB care [1]. The country has identified finding the missing TB cases as the single most important priority for TB control for the upcoming years, as each untreated case can infect 15–20 persons per year [2].

In terms of health financing, Nigeria spent 3.76% of gross domestic product (GDP) in 2017 [3]. With a per capita GDP of USD 5864, 8 and 32% of the TB budget was domestic and donor funded respectively. This leaves 60% of the National TB budget required to implement the Stop TB Partnership’s *Global Plan to End TB 2018–2022* unfunded [1, 3, 4]. In addition, 71 % of TB patients faced catastrophic health costs in 2017 [1].

The healthcare system in Nigeria is provided by the public healthcare system and an unregulated private sector [5, 6]. The public system is divided into primary health centres (PHCs) supported by the local government areas, secondary hospitals supported by the State governments, and tertiary hospitals supported by the Federal Government [7, 8]. The PHCs were put in place to serve as the first point of contact with healthcare for communities, but have been largely abandoned, both by the local governments and the communities they serve [7, 8]. Most of the available DR-TB care are in the public secondary and tertiary hospitals [9].

South Africa and Zimbabwe are two examples of countries also classified as high burden for TB, DR-TB and HIV in Africa. In 2017, South Africa spent 8.1% of its $13,396 GDP (PPP) on health and funded 87% of TB budget internally, with 0% unfunded. The country had 100 and 87% DR-TB notification and treatment rates in 2018. Comparatively, Zimbabwe spent 6.6% of $2782 GDP (PPP) on health and domestically funded less than 1% of its TB budget; with 31% donor funding, leaving 69% unfunded. Despite this, Zimbabwe had 27 and 25% DR-TB notification and treatment rates [1, 3, 4]. These suggest other barriers to TB care in Nigeria, in addition to health financing.

Equity is fairness or justice [10]. The WHO defines *equity* as the absence of avoidable social, economic, demographic or geographic differences among groups of people [11]. When applied to healthcare, most definitions of equity include health resources, how these resources are allocated, or align with the needs or characteristics of populations [10, 12–15]. However, equity in health care does not always translate to, and must be differentiated from, equity in health [16, 17]. Integral to these definitions are two key components- access to healthcare resources and the characteristics of individuals or populations [10, 13–15, 18].

Equitable health systems ensure services are available to everyone in need [12, 15, 19–21]. Policy experts have proposed that governments particularly evaluate health systems through their impact on the poor, in order to reverse the inequities in delivery [20, 21]. In the TB context, several authors including the WHO, have highlighted the need to target specific sociodemographic groups identified as being at a higher risk of contracting the disease or of having poorer access or outcomes, once infected [15, 22, 23]. Another aspect relevant to an equity analysis is the complexity of the TB care pathways- the number of patient visits and pre-treatment processes needed in order to achieve an outcome.

Access to healthcare is the ability to engage in timely use of the healthcare services that preserve or improve their health [18, 24]. Access to healthcare is both opportunity and ability to obtain needed health services, without risking financial hardship [25]. It can be defined as the possibility to identify needs, seek services, reach resources, obtain or use services, and be offered services appropriate to the needs of care [14]. Although difficult to measure, equal access to healthcare is defined by equal opportunities to access and utilize healthcare for those in equal need, resulting in equitable health outcomes [13, 26]. Using health system and individual-level data to identify areas of inequity, and with what factors it is associated, can be a more pragmatic approach than having a singular overall measure of how equitable a particular healthcare system is [26].

Equity and ease are two ways of looking at access to healthcare. Equity of access focuses on the health system, or supply-side, to ensure equal services for patients in equal need [27]. Ease of access explores individual and societal barriers to available healthcare services [14, 27]. These individual or societal barriers to access are inextricably linked to their social, economic, geographic or demographic characteristics [13, 14]. Effective and equitable access combines these two aspects: the ability to obtain timely health services based on needs, irrespective of sociodemographic characteristics, and without risking financial hardship [14, 18, 24, 25]. Both are important access indicators to monitor the performance of healthcare systems [18, 28, 29]. These definitions are in line with the Levesque et al. characterisation of access as having two main domains [14].

This study aimed to explore patient-centered ease and equity of access to diagnosis and treatment initiation for DR-TB patients through an analysis of policies, structures and processes for DR-TB care in Nigeria.

## Methodology

We used a transformative study design using key informant interview, focus group interviews, and document review. This is part of a larger mixed methods study, with previously published quantitative results [30]. In this paper, we report on the qualitative portion of the data. We used a transformative design which involves a theoretical lens to guide interpretation and advocate for action [31, 32]. Transformative research advocates for social justice and addresses power imbalances, by focusing on inequalities and marginalization, and this is reflected in every stage of the research [32, 33]. We focused on understanding the perspectives and experiences of patients (including those not on treatment), their relatives, care providers and program managers, through an equity of healthcare access framework. Our framework, adapted from the work of several authors [14, 34, 35], helped to identify inequities within the supply and demand sides of access, and highlight areas for improvement.

### Conceptual framework

The predominant theoretical framework guiding our transformative study is the Levesque patient-centred access to healthcare framework [12, 14, 19]. We chose the Levesque framework as we identified from our initial literature review, published elsewhere [36], an interplay of factors, both between the health system (supply) and patient (demand) levels, and at different stages of care. The framework also allowed us to explore some dimensions of the quality of care (patient-centred, equity, accessibility) [37, 38], which some authors have called the missing link in TB care [38, 39], without losing focus on the supply and demand dynamics of access.

Levesque et al. [14] puts the patients’ needs at the centre of the healthcare access framework, by focusing on the actual process of seeking care, including the various stages patients go through to actually receive needed care, and the abilities patients need to interact with health services [14]. At its core, the Levesque framework conceptualizes accessibility in five dimensions: approachability, acceptability, availability, affordability, and appropriateness. These dimensions must be matched with five corresponding abilities in patients: ability to perceive, seek, reach, pay, and engage, respectively [14].

**Fig. 1:**
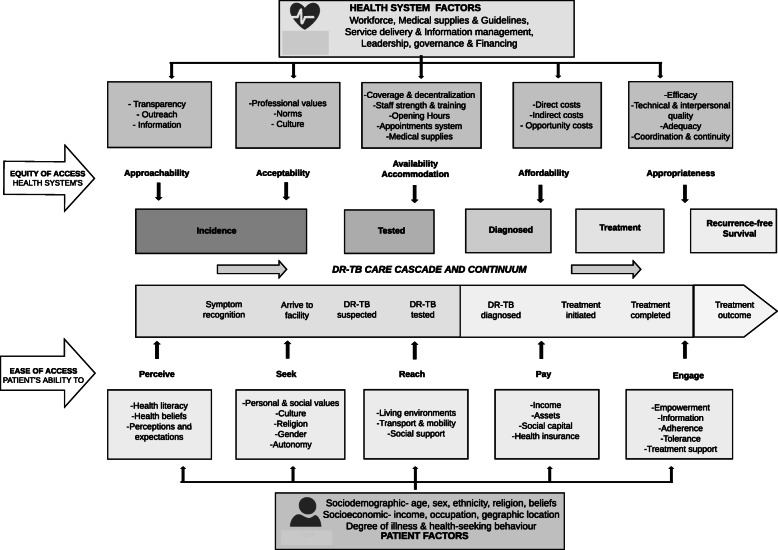
An adapted framework of equity and ease of access to DR-TB care

This was also considered through the lens of the continuum of care in health [34, 40, 41], the TB cascade of care [34, 42] and the WHO’s health system building blocks [35]. Our adapted framework (Fig. 1) was used to frame our qualitative instruments and to interpret findings.

At its core, the Levesque framework conceptualizes accessibility in five dimensions: approachability, acceptability, availability, affordability, and appropriateness of providers, organizations, institutions and systems. These dimensions must be matched with five corresponding abilities in patients for patients and communities: ability to perceive (or identify needs), seek services, reach resources, pay, and engage, respectively [14].

These healthcare and patient dimensions should be progressive as the patient moves from one stage of the care continuum to the other.

### Study population and data collection

We reviewed several National TB and Leprosy Control Program (NTBLCP) and Federal Ministry of Health (FMOH) policy and guideline [43–45] documents including the workers’ manual [46] and the 2015 annual TB program report [9], some unpublished program data [47–49] and WHO country profile [1].

**Fig. 2:**
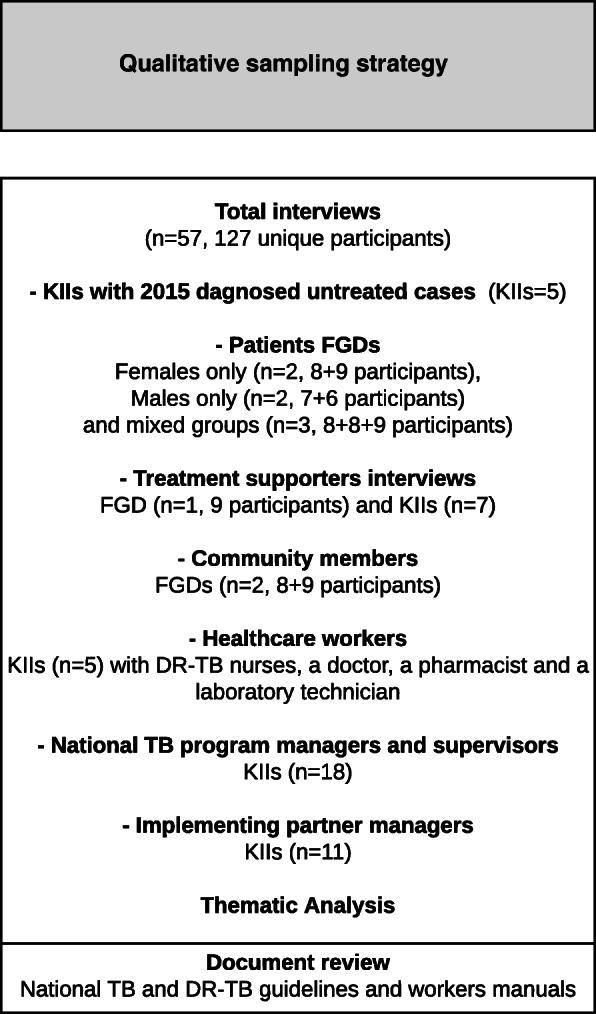
Ease and equity of access to free DR-TB care in Nigeria

This is part of a larger mixed methods study, with previously published quantitative results [30] and mixed methods results [50]. We analysed data from 57 interviews. These included focus group discussions (FGDs) (*n* = 10) with a total of 81 patients on treatment, treatment supporters and community members; as well as 46 in-depth interviews with untreated patients, healthcare providers and program managers. Interviews covered 4 locations in Nigeria including Ogun, Plateau, and Benue states and Abuja, Nigeria, between August and November 2017. Our sampling frame is shown in Fig. 2.

Our overall sampling strategy was purposive [51], selecting participants whose views would most likely be information-rich about delays in DR-TB care, as described above. Within this sampling approach, we selected participants based on availability and consent.

Healthcare workers’ questions included program structure, challenges and strengths, as well as their perception of access barriers and facilitators. Patients and their treatment supporters were also asked to describe barriers and facilitators to accessing DR-TB care that they, their relative or someone they knew had experienced. Female and male only FGDs were additionally probed for any particular challenges facing their gender in accessing care. In addition to the questions for healthcare workers, program managers and implementing partner respondents were asked about the available resources at the national, regional and state level for DR-TB care, their perspectives on the adequacy of these resources, and relevant policy documents on DR-TB care. The documents and guides recommended by respondents were also included in the analysis.

Informed consent from each participant were written or verbal (where needed), before each interview. The first author, who had prior DR-TB implementation experience in Nigeria, carried out the interviews with help from a field assistant. All interviews were conducted in English. However, some questions were translated into Nigerian pidgin, Yoruba or Hausa, if any participant requested it, using local translators. Interviews were audio-recorded, translated where needed and transcribed. Where portions of the transcripts included translations and responses in the other languages, a transcriber fluent in this language was used. We conducted member checking as a way to strengthen the rigour of our study [52]. Transcripts were sent back to all participants who had earlier agreed to be contacted for accuracy checking, some of whom responded with revised transcripts which we used to replace the original transcripts.

### Data analysis

Data analysis began during data collection to enable exploration and comparisons of new themes. Our interview guide, which was based on our initial literature review and conceptual framework, was used to deductively develop a coding tree before the process of coding began. Transcripts were checked and read through to give a general understanding of the data. The first phase of coding was inductive to allow new themes to be added to the coding tree. Codes were then deductively matched to the coding tree, with a few new codes added as needed. Coding of documents and interview transcripts were around themes based on our conceptual framework of Equity of access to DR-TB care (Fig. 1). The thematic analysis focused on how the system facilitates patient progression after arrival with symptoms at the TB clinic to the point of treatment initiation, as well as on patient pathways to care.

Our document analysis, which used content analysis, helped to triangulate evidence from the in-depth interviews. This also facilitated member checks (participant feedback on emerging themes) to ensure fidelity with participant intents as recommended by Seale [53]. Data analysis was facilitated by the use of the Quirkos software, version 1.6.1. Our findings are reported according to the consolidated criteria for reporting qualitative studies (COREQ) [54].

#### Ethics

The National Health Research Ethics Committee of Nigeria (NHREC/01/01/2007) and the Research Ethics Committee (CER) of the University of Montreal Hospital (17.060) granted ethical approval for this study An additional ethical approval was obtained from the Research Ethics Committee (CER) of sciences and health of the University of Montreal (CERSES-19-098-D). All participants gave written or verbal informed consent.

## Results

Our findings focus on the outputs needed from the health system and the patient to achieve DR-TB cure, by looking at required supply and demand dimensions and how they align with each step in the care continuum, starting from symptom recognition, through health-seeking to completing treatment. The five paired supply and demand dimensions, based on the Levesque framework of patient-centred healthcare access, is summarised in Fig. 2.

### Approachability and ability to perceive

Certain attributes of the healthcare system and of patients align when the patient recognizes that observed symptoms require medical attention and that certain health services can be accessed. The health system enables this through patient education, transparency and outreach services information. This stage in the care continuum should end with the patient deciding to seek care for their health problem.

The national guidelines stressed the need for patient education and community awareness and outreach activities. Routine patient education is to include cause and symptoms of TB, availability and free cost of treatment, where to seek healthcare, and how to prevent spread. These activities are to be implemented through home-based care for HIV and TB patients. The guidelines encourage healthcare workers to conduct campaigns and sensitization activities to increase testing requests, actively search for cases within the health centers especially for HIV patients, sensitize providers and engage community-based organizations. However, the guidelines are not immediately clear about the frequency or funding provisions for these campaigns. It also does not say how active case-finding in the community should be done, although several implementing partners are tasked with these activities.

Patients, their relatives and providers agreed that there was limited awareness about DR-TB in the communities.

*“The point is, not everybody knows about TB … I didn’t actually have the full knowledge of what TB was, so when I got infected, that was when I knew. Ah, this thing is really serious! But … people out there, I don’t think the information has been passed enough to people, especially those in the rural areas...they just feel it is this strong cough that doesn’t go away. [A herbalist] prescribes herbs for [them] to take and they think okay, it is just a normal cough that will [go away]....I think the awareness [is important] because when someone is aware [of] what is at stake … [they] will be … like... okay, this is [serious]...lets go to the hospital”* (Mixed patients FGD).

This often resulted in prolonged pathways to DR-TB care, because DR-TB care were most often available in the public sector. Most of the patients in our focus groups said they encountered these delays, mostly because of wasted time in the private sector. These delays ran from 3 months to 6 months, in a majority of cases, to more than 3 years, in a few cases, or even death. Patients acknowledged that the private sector, including patent medicine vendors (PMVs), community pharmacies and private hospitals, were the first point of contact with healthcare but that private practitioners had lower index of suspicion for DR-TB, and almost never referred them to the public sector. This apparent disconnection between the private sector and public sector care, resulting in misdiagnosis and mismanagement, threw patients into a sometimes-tragic search for a cure.

*“So, I [didn’t] really know what to do [anymore] I was … given the herbal [preparation], I went to church, I went to mosque, went to everywhere, but all [remained] the same I won’t lie to you, [DR-TB] is very strong and very powerful … I [also] went to a private hospital for 30 days treatment … they gave me some drugs to use, but the major [drug] they said I should be using is rifampicin. I [used] rifampicin for like two to three years. … When the result [of the drug susceptibility test] came out, … I was even surprised they wrote rifampicin [resistance] there, and … it was true I was [ab] using rifampicin*” (Diagnosed untreated male patient).

“*Private hospitals tell [patients] they don’t have [TB], my younger one that died, we [spent money] for the ... test, and they still insisted that he did not have [TB],* (Patients FGD)”.

“*He was driving [a truck] before, … [so] he stopped working … I was the one taking care of him at the Hospital, I [also] stopped my work … He [took herbal preparations], went to [the] pharmacy to buy medicines for himself, went to every possible place, there was no cure … He went to prayer houses too. Then he went to the hospital … the sickness was on and off. He will recover and fall sick again, that was why we kept going back … He has been sick for like 6 months [before starting treatment] … The sickness had already taken over his body, that was why [he died]*”*,* (Wife of patient who died a few months into DR-TB treatment).

The Nigerian Annual TB program reports also showed much lower coverage of free DR-TB services in the private sector, limiting the approachability of DR-TB healthcare.

Respondents mentioned that being told by a healthcare worker in the community, or by a former TB patient, helped them to realise their symptoms were treatable for free in the hospital. Healthcare workers were frustrated that there was more knowledge about HIV in their communities than of TB.

Government TB control officers and program managers mentioned that outreach activities have increased case finding in the communities they supervise.

*“So, somebody who would have stayed at home using traditional medicine, thinking that this is just an ordinary cough … but with the outreaches, [any] cough of 2 weeks … please come out for testing, and from the outreaches a lot of cases have been identified”* (Male program manager).

### Acceptability and ability to seek

For a patient to utilize healthcare, the health services need to have a higher perceived benefit than other options available to the patient, as well as not to violate any cultural, religious or social norms the patient has. This stage is also affected by the health systems professional values, culture and norms, as well as the patient’s autonomy. This stage ends with the patient choosing a particular source or type of care over other options.

As part of the TB private-public partnership strategy, the TB program worker’s manual included notes to organize regular meetings with relevant stakeholders including PMVs and traditional healers at state levels, with national oversight. Monitoring meetings with community-based organizations were also to include religious bodies. It was not clear from our data how often and where these activities were happening.

Patients, relatives and providers gave several narratives of patients visiting multiple sources of alternative care in search of a cure like PMVs, traditional healers and prayer houses, and most often before ever going to a health center. Sometimes, this was due to being unaware of DR-TB services, inconvenience of these services, misdiagnosis in a private hospital or family influence on patients’ autonomy.

*“I have not really seen many [cases like this] … except for a case of a student I saw when I went for on a supervisory visit … this student … was diagnosed with TB but his mum did not believe that he had TB. His mum felt that it was a spiritual issue that should be [handled] with prayers. I took up the phone and called her and despite all my pleading and explanation, [and even though] she said ok she was going to bring her son the next day, … she never came. … .This is one clear example of where a parent of a … minor prevented the child from having access to care”* TB Program Manager Interview*.*

The documents we reviewed did not mention protecting the right to health for minors, marginalized groups or other persons who might not be able to take a health decision on their own.

Other times, it was because the patients had more confidence in alternative healers than in the public sector hospital or wrongly attributed TB to other causes. As one HCW puts it:

“*I’ve treated one educated person here, [not] until he started the drug and [in] the second month [when] he [could] see the difference that … he believed truly TB is a disease. [Before then], he believed somebody [evil was harming] him; [imagine that] an educated [person]! So I [had] to make copies of … our National [training guides], to give to him, to go and read, that TB is [not supernatural]; and that you [can] be infected [anywhere], … from an infected person*” HCW interview.

Patients reported that they preferred to go to private hospitals because of widespread perception of better provider attitudes. The major acceptability barriers to public hospitals included poor provider attitude, unsanitary conditions, and lack of essential medical supplies and accountability.

*“Most of private hospital they don’t know [the right thing to do], and private hospital is where most people go to. [Government should] first … do something [about] private hospitals because … people will think [they] are getting [good treatment], whereas it is just [the wrong medication] … In this country, if something happens, people … go to private hospitals, and they should. [Government hospitals] don’t treat people well. I can’t give birth in a government hospital , my younger one was dying in a government hospital and they said it is not their business, if you don’t get a particular [item], if you don’t get [say, a facemask], they will not attend to [your] child, before we could get [the facemask], the child had died. Government hospitals treat people like dogs, like animals, like … whatever happens to you is not their business, they will [still] get paid. In private hospitals, they will be sweeping and cleaning every minute … , and telling you sorry every time, [and] you … feel a little bit consoled”* (Female patients FGD 1).

Patients were more inclined to use the public sector on the recommendation of someone in their community that they trusted, like their pastor or family member. Interviews with members of the community also highlighted the effect of community awareness campaigns in changing people’s beliefs about health services.

### Availability, accommodation and ability to reach

For patients to be able to reach a health service, it needs to be geographically available, with accessible opening hours and appointment systems. The patient should have access to secure transport to reach these services. At the health facility, the patient needs to come in contact with a knowledgeable provider who suspects and tests for DR-TB. This would also need to align with the patient’s support system. At the end of this cascade stage, the patient should be known to the health system and recorded as “tested”.

Based on the annual National TB program reports and data from 2015 to 2017 that were part of our document review, coverage in services was scaled up nationally: testing facilities increased from 201 to 386 (testing), in-patient treatment centres from 13 to 29 and community outpatient treatment centres from 5 to 200. Most of the testing facilities were located in the tertiary and secondary public health centres, with only13 and 6% of testing in the private sector and primary health care level respectively. In-patient treatment centres were in 27 out of 37 states, and all were in tertiary or secondary facilities, with 17% in private hospitals, excluding patients who were initiated on treatment in the communities. Geographic coverage of testing was 48% at the end of 2017.

Respondents mentioned the lack of testing and treatment facilities near them as a barrier. ﻿ Many patients lived far from the health facility, with transportation difficulties, especially in rural areas.

*“Some people do not have the opportunity to come down to this place; if it is in their State they will also be able to go to the clinic close to them to [test], knowing that when they get there they will [be treated] well”* (Female patients FGD 1).

Several health system barriers were noted including clinic and laboratory operational delays, data errors and stock-outs of essential health products. Healthcare workers gave instances of patients giving wrong contact information, due to poor confidence in the public healthcare system, which affected patient tracking and resulted in loss to follow-up. The treatment guidelines and workers manuals we looked at did not include any procedure for address verification for patients being tested or initiated on treatment.

Healthcare workers also noted limited staff numbers as a major challenge.

*“... because we don’t have manpower on ground. … In a particular facility probably, they are only two [staff] and in some cases there is only one personnel. Now you will be handling this, … you will be doing this [and that]. So, at the end you may not even have time for some of your patients ... that is the greatest challenge we have”* (Female HCW)

### Affordability and ability to pay

The direct and indirect costs of accessing care and the patient’s socioeconomic situation determine whether a patient gets diagnosed and placed on treatment. These costs and ability to keep paying for them will determine if the patient initiates and continues on treatment.

Patients narrated facing catastrophic treatment costs, mostly in the private sector, before finding the right health center for DR-TB care. Other direct and indirect costs were related to transportation for follow-up appointments and for pre-treatment investigations.

However, with support from partners, the TB program pays transport and social support to patients enrolled on treatment, at approximately USD100 per month [48]. Patients and their relatives repeatedly mentioned that the financial support was the biggest facilitator of access for them.

*﻿“We thank the sponsors of [this] program because if not for them, things would have gone wrong. Because so many [people] didn’t go for [the] injections because of money, so many [people didn’t] have money for [transportation], feeding but we thank [the program]. If not for the money [they] gave us, many [more] would have died. Things would have gone [very] wrong [for us] but we thank them …*. *Thank you very much.”* (Treatment supporters FGD).

*“I used to hear that they heal people with [TB here], but I was like - how much will I [need to pay when I get here]; but when I heard it was free, that was what gave me the opportunity to come here”* (Female-only FGD)*.*

### Appropriateness and ability to engage

The healthcare system also needs to be efficacious, well-coordinated, uninterrupted and support the patient to be empowered and adhere to the treatment regimen to its completion. The patient also needs to be able to tolerate or withstand the effects of treatment and have adequate support from their social network. Only then can the desired treatment outcome be achieved.

There were instances of patients losing hope during the long duration of treatment or having unbearable side effects, including pain from injections, to the point where the possibility of dying was preferable to remaining on treatment.

*“[An elderly man], … when he sees the tray for drugs, … he will start vomiting, ha! even when they have not given him, as soon as he sees it, he starts vomiting. At last even nurse or doctor, when he sees them he will just start vomiting, and finally he said he wants to go, … if he even sees the color of the doctors or nurses uniform he will be so afraid … he said it is better for him to go and die … maybe he is dead but we don’t know, but if he just sees them even there is no drugs he will start vomiting. .. they gave him paper to sign out of treatment … and he signed out and left because he [couldn’t] bear the pain [anymore]”* (Female patients FGD 2).

“*I lost hope...﻿It got to a point, I was throwing my drugs away, because I just told them they should leave me to [die]... I was just so tired of everything*” Patients FGD.

“*We have some that after starting treatment, due to adverse drug reaction you know, some of them may tell you that I better die than to be taking this drug*” HCW interview.

In several cases, having a caring healthcare worker made the difference between continuing treatment or not.

“*Taking the drugs, to be frank, is very difficult. When you take the drugs, you will feel serious pain. Sometimes some people will be shouting, shouting; … the injection is like that. … sometimes we are together with the nurses while the injection is being given, and during that period, you will be see different people shouting, shouting because of the injection, some people will even be running from the injection. To be frank the nurses are very caring. and God will bless them all for their effort*” Female Patients FGD.

“*After some months, I saw the next [medication], I refused because the pain [I was] going through. So, I didn’t want to take the treatment, but the way [the HCWs] talked, pleaded. When they came, they started pleading with me … That’s why I … decided, let [me just] go ahead and take it*” Mixed Patients FGD.

Healthcare workers cited a few instances of patients whose families prevented their treatment completion because of their own beliefs that the private sector will offer a cure.

At the health system level, several barriers were noted, including stock out of essential supplies, and inadequate patient counseling.

### Overview of equity and ease of access to free DR-TB services

Our document review found, and providers and the patients affirmed that the DR-TB program provided financial support for patients on treatment, which patients acknowledged as the main facilitator to access.

“*The day they gave me the result and said it was TB, I was like ah! And I started thinking that where do I … get money [for treatment]. I told my husband and he was worried. The doctor then said that whatever we are using here will be free of charge. I was like, it a lie, there is no [way] I will come here and will not spend money. I wanted to drop the money on me. They were like, no, I should go [home] with it.... When we got to where we will do [the] X-ray, I [asked], how much am I spending? They said I am not spending anything, that I should go home, because everything they do here is free.*” Patients FGD.

There were very many instances of healthcare workers personally going above the line of duty to help patients remain in care.

“*We pay nothing. Even when I go to hospital without money, the person giving us drug [would] give me money for transport from her personal purse and I keep thanking her till today because she really took good care of me*” Patients FGD.

However, to be placed on treatment, there were significant challenges with coverage of services, prolonged care processes, operational errors and provider attitudes.

*“When I got to that general hospital to do test … , which [was 6 months ago] they said … am going to do eight tests, am going to do seven there [at the general hospital] then the other one at [another location]. [eventually], I did everything, I have [done] the … seventh test at general [hospital] … so it is remaining, one, at [the other location], they said that one … audiometric or (hiss) I have forgotten what the doctor called it. … which is the eight one. … [I just did], the x-ray. Everything -the results- are still at home”* (Untreated male patient).

All groups of respondents mentioned that some groups of patients had more difficulty accessing care. These include patients living in rural communities far from TB healthcare centres, children whose parents had low trust or information about public healthcare, patients in the private sector, women due to adverse cultural norms that necessitated asking for their husbands’ permission to access care, workers and students.

These difficulties are reflected in the following quotes:

*“A 12 years old [girl] came down with … resistance and the mother vehemently refuse to take her for treatment all in the name of she has given her some cough syrup. The state team went there … yet this woman stood her ground that she will not allow the daughter to leave … The TBLS (TB Local Government Supervisor) … the woman took one knife at … him … So, now the small [child] that is bearing the pain. But because she is small, she can’t take decision on her own.”* (Male program manager).

Livelihoods and education were threatened or interrupted, even for patients’ relatives, in order to navigate the process of care.

﻿“*I was learning before, I should have finished learning this year before this TB stopped my learning. It was remaining 4 months for me to complete*” (Female patient relative).

*“The reason why I don’t want to start now is that … is work! My work … And my house rent is going to be due in November which is next two months; … the reason why I don’t want to [go for treatment] now, is that if the house rent should be due [how can] I tell my landlord that I am leaving for the hospital?! ...I am going to pay for the house rent*” (Untreated male patient).

Overall, there was cohesiveness between data source (document versus interviews) and respondent type. However, there were a couple of differences. For example, while the national guidelines recommended that community awareness be carried out, it was not very clear how these were to be funded or implemented by the healthcare worker. Also, several healthcare workers cited cases of female patients and children being prevented from accessing care because of an authority figure, participants in the female-only FGDs did not mention this, even when probed specifically for this. However, a phone interview with a female adult patient living with her father, was interrupted by the father, who asked that his daughter never be contacted again by the DR-TB program as she was already healed by prayers.

## Discussion

We conducted qualitative interviews in 4 Nigerian states, combining this with documentary review of guidelines and policies in place within the DR-TB program, in order to explore barriers to access along the pathway to care. Our findings highlight gaps in equity of access to DR-TB healthcare, in terms of approachability, acceptability, availability, affordability and appropriateness. Overall, our results agree with most of the findings from other sub-Saharan African countries, detailed in our previously published systematic review [36].

Our study identified several barriers within the current DR-TB health system that impede equitable access along the pathway to care for different groups of patients. We identified access to information about TB in general, and about the availability of free services in particular, as a major challenge, preventing engagement with the system and leading to prolonged care pathways. A study in Nigeria on health-seeking pathways of TB patients found that the perceived cause of TB influenced their first choice of treatment [55]. Patients who believed TB was caused by witchcraft were more likely to use alternative treatments. Many participants in our study observed that patients only go the hospital after exhausting other options in the private sector. Treatment delays were, thus, related more to inadequate community awareness about TB disease and available services, and distrust of the public sector, agreeing with several studies in sub-Saharan Africa [56–58]. Approachability in several SSA countries were hampered by poor provider knowledge and attitudes, poor referral systems and patient tracking, as in our study [57–68].

The lack of referrals between the private and public sector in our study has been found in other studies [59–61]. This was identified as a major missed opportunity for DR-TB care, as the private sector remains the first point of healthcare contact for many TB patients [5].

Poor approachability and ability to perceive often resulted in patients not being accepting of available services, preferring alternatives. This has been highlighted in other SSA studies [56, 57, 69, 70].

Inadequate coverage of diagnostic and treatment facilities, as well as the poor availability of health products and other operational challenges is well documented in studies from SSA [56–58, 62, 65–68, 70–79]. Our study agrees with these findings.

A number of studies have identified the indirect costs of care including paying for transport to health facilities, and other high opportunity costs borne by patients [57, 68]. Participants in our study mentioned repeatedly mentioned these costs, which were alleviated by the financial support provided by the program.

Our study also identified barriers to access that are not so common in literature – where family members impeded patients’ care, particularly women and children, due to their own distrust in the health system or preference for alternative care.

As a limitation, we were not able to corroborate that all the documents reviewed were sufficiently available to field staff, and how much these documents influenced their practice, and it might be necessary to evaluate this effect in future research. The major documents with which most of the healthcare workers were familiar were the TB guidelines and workers’ manuals. Secondly, the purposive sampling method we used may have introduced selection bias.

Another limitation is the fact that some of our transcripts included responses in other languages that had to be translated. We were not always able to receive feedback from these respondents on the accuracy of our translations.

### Recommendations

In line with our transformative research approach [32, 33], we emphasize possible interventions to enhance ease and equity of access, based on our findings. In order to improve case finding and treatment coverage rates, the TB program in Nigeria needs to focus attention at the different contextual and health system factors impeding access.

Several community health awareness interventions across different disease settings have documented effectiveness, and these include ‘edutainment’ using media (radio, TV and print), school and community outreaches, dedicated community helplines in local languages [80–86]. These messages need to be tailored to context and culturally sensitive to be effective and will go beyond the current strategies employed by the Nigerian TB program, which include patient educational posters on the walls of facilities and periodic active case finding in communities. The current strategies rely on a TB patient presenting voluntarily at a facility before learning about available services, or through targeted active case finding activities by implementing partners.

Another opportunity for improving equity of access is improving the referral system between the private and public sectors. Nigeria has one of the highest percentage of patients using the private sector as first point of care (66–92%) in the world, with the over 60,000 PMVs in the country [5]. Currently, the TB program has mechanisms in place to meet with representatives from community-based organizations, including traditional and religious organizations, and there are implementing partners working on improving private sector engagement [5]. However, these efforts have not translated to significant improvements in referral between the private and public sector [5]. Current fees-for-referrals from private facilities will need sustained funding, and additional research is needed to find out more about these programs, including coverage and whether information about the scheme is available to all PMVs and private hospitals. As suggested by the respondents themselves, we agree that media engagement is an opportunity to increase public awareness using culturally adapted media programs in different languages to take the information directly into communities where it is most needed.

An immediate step for the National program could include additional address verification for all patients being tested for TB. The TB and DR-TB guidelines currently do not include instructions to public providers on address verification steps, unlike in the South African and Zimbabwean guidelines [87, 88]. Verifying contact information, especially in settings with informal address systems, has potential in reducing loss to follow-up and improving contact tracing [89–91]. One possibility might for the TB program to ask patients to provide contact verifiable addresses for their treatment supporters on testing to serve as an additional way to contact patients if they become lost to follow-up; this ‘guarantors’ or ‘referees’ system has been used successfully by financial institutions in Nigeria and similar settings to recover bad loans [92–95]. However, operational issues will need to be monitored and addressed to ensure that the benefits outweigh the costs of this additional task in an already overburdened system.

Compared to guidelines from South Africa and Zimbabwe [87, 88], the guideline from Nigeria would benefit from the inclusion of clearly delineated timelines such as replacing “early diagnosis” with “diagnosis of DR-TB within 48 hours of submitting a sample” and “timely treatment initiation” with “commencement of TB treatment within five days of reporting to a health facility with symptoms of TB”. The Nigerian guideline could also benefit from the inclusion of relevant sections from the Nigerian Health Act that give weight to patient’s rights or highlight penalties for endangering the health of others by refusing care or preventing them from accessing care. This would protect the rights of women and children identified in our study as having limited autonomy to access care.

In our literature review published earlier, we summarised recommendations made by the authors to improve the barriers to access by paired dimensions [36].

## Conclusions

DR-TB services in Nigeria are not always equitable, and patients face significant barriers to care. Our findings highlight health system barriers around coverage, operations errors, and provider attitudes, with patient financial support as a major facilitator; and patient barriers of awareness, perceptions of poor public sector care, beliefs and preference for alternative care. We discussed several opportunities for improvement to the demand and supply factors impacting access to DR-TB healthcare. Given the urgent need to increase notification and treatment coverage, there is a need for the TB program to innovate and reduce these barriers as well as adapting to the needs of the patients, including improving referral system with the private sector, community awareness, and protecting the rights of patients with limited autonomy.

## Data Availability

All data supporting our findings are included in this published article.
